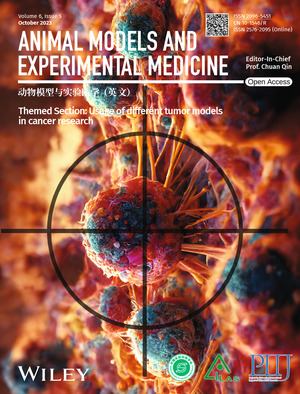# Cover Picture

**DOI:** 10.1002/ame2.12246

**Published:** 2023-10-30

## Abstract

The cover image is based on the Themed Section of this issue: Usage of different tumor models in cancer research. The research on the mechanism of cancer occurrence and metastasis has always been an international hotspot. This section focuses on the progress in tumor research using animal models and organoids.The source of the background of the cover image: Adobe Stock Number: 535775715 © auntspray/Adobe